# Response Latency Tuning by Retinal Circuits Modulates Signal Efficiency

**DOI:** 10.1038/s41598-019-51756-y

**Published:** 2019-10-22

**Authors:** Ádám Jonatán Tengölics, Gergely Szarka, Alma Ganczer, Edina Szabó-Meleg, Miklós Nyitrai, Tamás Kovács-Öller, Béla Völgyi

**Affiliations:** 1 MTA-PTE NAP-2 Retinal Electrical Synapses Research Group, Pécs, H-7624 Hungary; 2 János Szentágothai Research Centre, Pécs, H-7624 Hungary; 3 Department of Experimental Zoology and Neurobiology, University of Pécs, Pécs, H-7624 Hungary; 4 Department of Biophysics, University of Pécs Medical School, Pécs, H-7624 Hungary; 5 Nuclear-Mitochondrial Interactions Research Group, Hungarian Academy of Sciences (MTA-PTE), Pécs, H-7624 Hungary

**Keywords:** Neural circuits, Retina

## Abstract

In the visual system, retinal ganglion cells (RGCs) of various subtypes encode preprocessed photoreceptor signals into a spike output which is then transmitted towards the brain through parallel feature pathways. Spike timing determines how each feature signal contributes to the output of downstream neurons in visual brain centers, thereby influencing efficiency in visual perception. In this study, we demonstrate a marked population-wide variability in RGC response latency that is independent of trial-to-trial variability and recording approach. RGC response latencies to simple visual stimuli vary considerably in a heterogenous cell population but remain reliable when RGCs of a single subtype are compared. This subtype specificity, however, vanishes when the retinal circuitry is bypassed via direct RGC electrical stimulation. This suggests that latency is primarily determined by the signaling speed through retinal pathways that provide subtype specific inputs to RGCs. In addition, response latency is significantly altered when GABA inhibition or gap junction signaling is disturbed, which further supports the key role of retinal microcircuits in latency tuning. Finally, modulation of stimulus parameters affects individual RGC response delays considerably. Based on these findings, we hypothesize that retinal microcircuits fine-tune RGC response latency, which in turn determines the context-dependent weighing of each signal and its contribution to visual perception.

## Introduction

In the mammalian retina, various features of the visual scene are encoded by dedicated retinal ganglion cell (RGC) subtypes and their spike signals are streamed towards the brain through parallel visual pathways. These separate information avenues inform the brain about object- and/or background motion^1–4^, orientation^5–7^, direction of movement^8–11^ and many other features. There is much evidence showing that RGCs detect stimuli with various time delays^12,13^, resulting in an uneven launch of signals through various visual pathways. Moreover, the dissimilar RGC axon calibers may result in additional differences in signaling speed towards the brain as well^13,14^. It has been found earlier that our visual system processes information about moving objects more rapidly than flashed objects and due to this latency difference, we are prone to well-known visual illusions^15–17^. These latter observations reflect that certain visual feature pathways operate faster than others and dissimilar RGC response latencies might provide the backbone for this phenomenon. A recent work presented evidence in the retina of the cold-blooded salamander that the RGC latency difference can be exploited by the visual system to encode certain spatio-temporal features of the scene^18^. These facts indicate that RGC response latency is a non-negligible factor in understanding the fundamentals of visual processing. However, some important questions yet remain unanswered: *(i)* how great is the latency range across the entire RGC population; *(ii)* is the observed range based on imprecise responding of RGCs or summated by RGC subtype specific subranges; *(iii)* are latency differences due to dissimilar RGC membrane properties and/or determined by the signaling speed through the upstream retinal circuitry and *(iv)* how does the visual system exploit differences in response latency to perfect mammalian vision?

To answer some of these cardinal questions here, light-evoked responses of mouse RGCs were recorded *via* multiple recording paradigms while stimulus parameters were modulated or pharmacological interventions were carried out. Simple full-field photopic light stimuli exerted RGC responses with rather different delays, with most of them ranging between 40 and 240 ms. Our results showed that response delays were RGC subtype specific and varied extensively when stimulus parameters were altered. Trial-to-trial variability of RGC light responses were also subtype dependent but contributed little to the observed population-wide latency range. Results of this study thus indicate that key components of a latency control mechanism are embedded in retinal microcircuits that fine-tune RGC response latency, thereby adjusting the weighing of signals in generating postsynaptic effects.

## Materials and Methods

### Animals and preparation

Adult (P30-90) C57BL/6J and Thy1-GCamP3 (JAX #029860) mice were maintained in a 12/12 hours dark/light cycle; all experiments were carried out during the day with dissections between 10–12 AM and after 12 hours of dark-adaptation prior to experiments. For one set of experiments a PV-GCaMP6f hybrid mouse line was utilized as well. This line was obtained by crossing *Pvalb*^*tm1(cre)Arbr*^ (JAX #017320) mice with the Ai95(RCL-GCaMP6f)-D (C57BL/6J) (JAX #028865) animals. Mice were deeply anesthetized with the inhalation of Forane (4%, 0.2 ml/l) and then sacrificed using cervical dislocation. Eyes and retinas were removed under dim red illumination and hemisected anterior to the ora-serrata. Cornea, lens, vitreous humor, as well as the pigment epithelium were isolated, and the resultant isolated retina was attached to a filter paper (Millipore). Specimen then were placed in a superfusion chamber mounted in a light-tight Faraday cage and superfused with an oxygenated (95% O_2_, 5% CO_2_) and heated (34 °C) mammalian Ringer solution (pH = 7.4). Animal handling, housing, and experimental procedures were reviewed and approved by the ethical committee of the University of Pécs (BA02/2000-6/2006; BA/35/51-42/2016). All animals were treated in accordance with the ARVO Statement for the Use of Animals in Ophthalmic and Vision Research. All efforts were made to minimize pain and discomfort during the experiments and all procedures were done by obeying the 3R law.

### Ca^++^-imaging

Ca^++^-imaging experiments were carried out in the Thy-1 GCaMP3 transgenic mouse line, in which the Ca^++^-indicator is expressed by all RGCs. In the PV-GCaMP6f line, GCaMP was expressed only by a subset (6 cell types^19^) of RGCs. A modified upright Nikon FN1 microscope equipped with 4x, 10x, 40x and 60x objectives (NA 0.31, 0.3, 0.8 and 1.0 respectively) was used for imaging. RGC responses were captured upon full-field photopic light stimulation (l = 490 nm, t = 3 s) and sampled at 33.3 Hz. We measured the RGC response delay and trial-to-trial variability. Excitation light was generated by Polychrome V monochromator (FEI/TILL Photonics; Oregon) and image sequences were captured by a Retiga 2000DC digital camera (QImaging) while the acquisition was performed with Live Acquisition software (FEI/TILL Photonics; Oregon). Alternatively, an Intensilight light source (Nikon) and a Sony A6300 camera were used for the image sequence acquisition driven by TTL-pulses, which were generated in the WinWCP (John Dempster, University of Stratchlyde) acquisition software. In this latter case, a magnetic switch modulated a LED-light source and the sampling frequency was 60 Hz with constant ISO and filter settings, in Full HD (1080p) resolution. In most cases, input/output signals were synchronized by an Instrument Control Unit (ICU; FEI/TILL Photonics Oregon). Image sequences were first handled in FIJI (NIH, Bethesda); region of interests (ROI) for examined RGCs were selected here by the ROI manager. Image analyses were performed in Matlab and NeuroCa^20^ integrated framework. Temporal ROI sequences of a network burst were implemented, nonparametric statistical analyses were performed and temporal orders were determined. Ca^++^-transients (spikes) were collected for all RGCs in the examined frame and initiation times, amplitudes and widths were determined by using fluorescence changes (ΔF/F_0_) in each ROI^20^. Some analyses on acquired imaging data were performed in Microsoft Excel and/or Origin2018 (OriginLab, Northampton, MA, USA).

### Extracellular electrophysiology

Unit extracellular recordings were performed from RGCs with carbon microelectrodes (1MΩ; Kation Scientific LCC Minneapolis, MN, USA) attached to an AC differential amplifier (DAM80i, World Precision Instruments) and digitized with an analog-to-digital board (Digidata 1440a; Axon Instruments, Sunnyvale, CA, USA). RGC action potentials were recorded with Axoscope (Axon Instruments, Foster City, CA) with a sampling rate of 20 kHz. In some experiments, multielectrode extracellular electrophysiology was also performed by utilizing either a 60 or a 120 channel MEA system (Multichannel Systems Gmbh, Germany) that allowed for recording simultaneously from many RGCs. Analyses were performed offline using Spike 2 (Cambridge Electronics Design Ltd., Cambridge, UK), Off-line Sorter (Plexon, Dallas, TX) and NeuroExplorer 5 (Nex Technologies, Littleton, MA) softwares. Histograms and graphs were generated in Origin2018 (OriginLab, Northampton, MA, USA). Collected spikes were timestamped (bin size 10 ms) to generate peristimulus-time histograms (PSTH; NeuroExplorer 5, Nex Technologies, Littleton, MA). PSTHs then served to obtain response peak positions and delays. Response fadeout time (PSTHτ) and trial-to-trial variability were calculated in Microsoft© Office Excel, further data- and statistical analyses were performed in Origin 2018 (OriginLab, Northampton, MA, USA).

### Patch clamp recordings, dye injections

Patch clamp recordings were performed with an Axopatch 200B Patch clamp (PC) amplifier (Axon Instruments) and ECS filled PC pipettes (~6 MΩ; borosilicate glass, 1.5/0.84 mm ID/OD, WPI) in a cell attached configuration (Voltage Clamp mode). Signals were digitized with a Digidata 1440A ADC (Axon Instruments) and acquired with WinWCP software (John Dempster, University of Stratchlyde). Electrodes used for dye staining and stimulation were filled with ICS (4–9 MΩ borosilicate glass pipettes, 1.5/0.84 mm ID/OD, WPI). Electrodes were pulled with a P-87 micropipette puller (Sutter Instruments). ICS contained (in mM) 125 potassium gluconate, 8 NaCl, 0.1 CaCl2, 0.6 MgCl2, 1 EGTA, 10 HEPES, 2 Mg-ATP and 0.4 Na-GTP at pH 7.3 (KOH-adjusted) and supplemented with 0.5% A568-hydrazide and 2% Neurobiotin to fill up target cells upon successful membrane-breach.

### Light stimulation

Full-field light stimuli were delivered by a green (λ = 525 nm) LED and light beams were directly focused on the surface of the retina. Stimulus intensity was expressed as the time-averaged rate of photoisomerizations per rod (Rh^∗^/rod/s). In order to obtain these values, light intensity was first measured with a radiometer (Ealing Electro-Optics, Holliston, MA) and then converted assuming an average rod density of 437,000 rods/mm^2^ ^21^ and quantum efficiency of 0.67^22^. In experiments where intensity response functions of RGCs were generated, stimulus intensity varied from 10^−2^ to 10^4^ Rh*/rod/s by using an LED driver (with adjustable DC output); in the rest of the experiments photopic stimuli (>102 Rh*/rod/s) were delivered to the preparation. For spectral sensitivity recordings, a 100 W tungsten-halogen lamp and a light bench was used to deliver full-field white light. Light intensity was adjusted by neutral density filters (between log−5.5 and log 0; log 0 = 0.25 mW/cm^2^) and spectral components were determined by a color bandpass filter set (330, 360, 409, 437, 472, 495, 527, 562, 590, 632, 645 and 647 nm).

### Pharmacology

Pharmacological experiments were carried out by recording in control conditions (mammalian Ringer’s) first and then switching to containers holding either 40 µM meclofenamic acid (MFA) or 50 µM picrotoxin (PTX) to block GJs and GABA mediated signaling, respectively. Recordings were repeated under drug administration and then wash-back incubations and recordings were attempted.

### Streptavidine staining

After Neurobiotin filling, samples were incubated for minimum 30 minutes then tissues were fixed in 4% PFA for 15–25 minutes, washed with PBS, blocked with BTA (5% Bovine Serum Albumin, 0.5% Triton X-100, 0.05% Na-azide in PBS), then incubated in streptavidin-Cy3 or -Cy5 (Thermo Fisher Scientific) in BTA for overnight. Washed retina samples were placed on slides mounted in Vectashield (Vector laboratories) and cover-slipped for microscopy.

### Confocal microscopy and image processing

Retinal samples were scanned with a Zeiss LSM710 confocal microscope with 20x (Z = 1 μm; Zeiss W Plan-Apochromat 20/1.0) and 63x objectives (Z = 0.5 μm; Zeiss Plan Apochromat 63/1.4) in high resolution and normalized laser intensity. Minor manipulations of brightness and contrast of images were performed in (Adobe Photoshop CC, Adobe Systems Inc., San Jose, CA).

## Results

### Timing of RGC light responses occurs on a broad range

**Figure 1:**
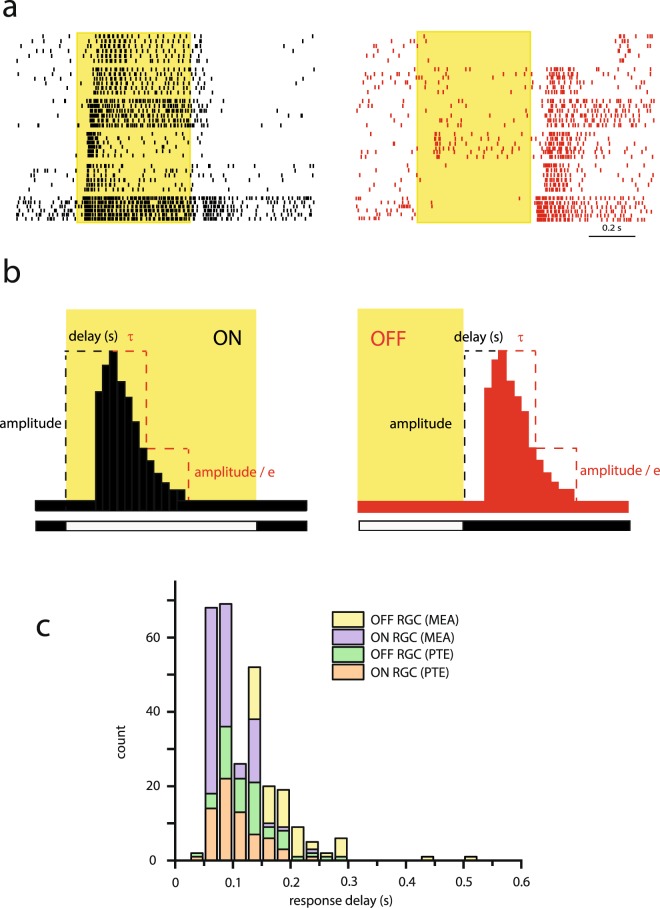
RGC Response Latencies Cover a Wide Range. (**a**) Perievent raster diagrams generated upon light evoked spike activity of 12 RGCs (6 ON-cells on the left and 6 OFF-cells on the right) are shown in an order of decreasing response delay. Each row corresponds to a different RGC recorded in a single experiment simultaneously. Yellow areas (here and in all following figures) reflect the on- and off-sets of full-field light stimuli. (**b**) Schemes explain how amplitude, delay and τ (for transiency) were determined from PSTHs generated upon ON (left) and OFF (right) light-evoked responses. Response delays were defined as the time elapsed from the light on- (ON-cells) or off-set (OFF-cells) to the PSTH peak (highest spiking frequency). The value of τ was determined as the time required for responses to drop to the *1/e of the maximum amplitude. (**c**) Histogram showing the distribution of PSTH peak delays for RGCs in either Tungsten electrode (n = 67 ON and n = 54 OFF cells) or MEA recordings (n = 107 ON and n = 52 OFF cells). The range of average delays was 50–280 msec.

To examine the time course of RGC signaling in the mouse retina, light stimulus evoked responses were recorded. In order to minimize potential methodology related artefacts (electrode bias, acquisition delay etc.), we performed spike recordings by utilizing either a Tungsten extracellular electrode or a multi electrode array (MEA; 64 or 128 channels; 100 μm electrode distance) and combined extracellular data were used for the following analysis. In a first set of experiments, RGCs were presented with six consecutive photopic (I = 977 Rh*/rod/s; Rh* here and throughout this work reflect rod isomerization) full-field TTL light flashes (duration = 0.5 s; cycle length = 2 s) and light evoked spike activity was recorded. Responses with either ON or OFF polarity appeared to show a wide range of variability in their latencies (Fig. 1a). To investigate the extent of this variability, peristimulus time histograms (PSTHs) were generated upon spike recordings and response delays were determined by calculating time-to-peak values (Fig. 1b). As ON- and OFF-signals were carried by separate retinal pathways to RGCs, ON and OFF response components of ON-OFF cells were handled separately. We found that RGC response delays ranged from 45 to 300 ms (Fig. 1c) across the recorded population, with only a few cells displaying even longer delays. When responses were sorted into ON- and OFF- categories based on response polarity, resultant response delays ranged between 45 and 275 ms for the majority of both ON- and OFF-cells. The RGC response delay distribution histogram peaked at about 75–100 ms with no clear sign for the separation of sluggish and brisk cells. Average response delays differed somewhat for Tungsten electrode recordings (ON: mean = 102.46, SD = 39.17, n = 67; OFF: mean = 124.44, SD = 49 ms; n = 54) and MEA recordings (ON: mean = 83.9, SD = 28.77, n = 107; OFF: mean = 197.16, SD = 71.46 ms; n = 52) as a sign of certain methodological bias but response delays fell in a similarly broad range for both recording approaches.

It seemed unlikely that the observed response latency variability stems from the dissimilar times currents require to travel from the recorded soma to the electrode because Tungsten electrodes maintained a direct contact with recorded somata and they showed results comparable to those of MEA recordings. To gain further proof of this, the soma-to-electrode travel time was calculated based on selected MEA recordings in which multiple electrodes (with different soma-to-electrode distances) recorded the spiking activity of the same cell (n = 7). We found that signal delays and electrode distances showed no clear sign of correlation, indicating that a variety in electrode distance is a negligible factor in our measurements (Supplemental Fig. 1). Therefore, soma-to-electrode distance could not account for the above described RGC response delay variability.

Rather than increased spiking rates, the delay of the first spike (first-spike time) upon stimulus onset may carry information about the stimulus^23^, indicating that spike timing rather than spike rate must be considered in this study. To that end, response delays (obtained from the same set of RGC recordings as above) were determined based on the timing of the first spike following the stimulus onset (the stimulus onset was used as a reference). We found that first spike time values in general were lower (ON-cells: 67.4 ms, SD = 43.3, n = 67 vs. OFF-cells: 91.18 ms, SD = 120, n = 57) than those of corresponding PSTH delays. However, delay distribution histograms appeared very similar in terms of the delay ranges for the two methods (Supplemental Fig. 2). This suggests that our observation on response latencies is independent of the utilized methodology and instead, is an inherent feature of RGC signaling. Therefore, all further analyses were carried out utilizing PSTH peak delay values.

### Variability in Individual RGC response delays

**Figure 2:**
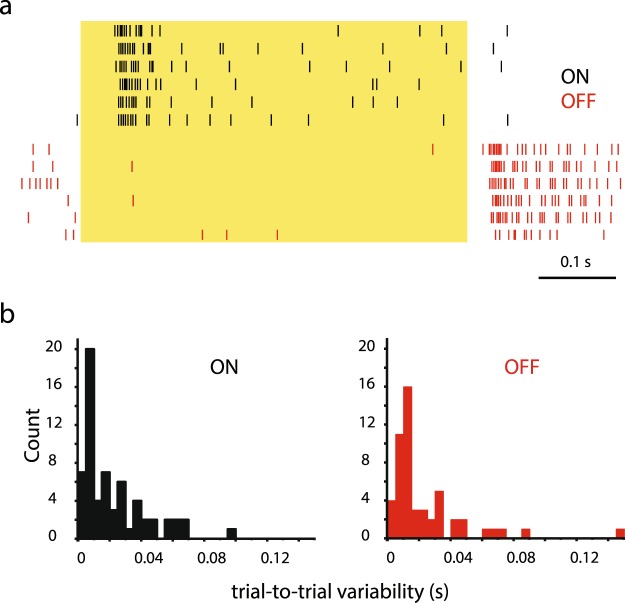
Individual RGCs Display no Significant Trial-to-trial Variability in Response Latency. (**a**) Perievent raster diagrams show six consecutive light responses of an ON (top) and an OFF (bottom) RGC. Individual responses display trial-to-trial variability in response latency for both cells. (**b**) Histograms show the distribution of the trial-to-trial variabilities of responses (6 trials with the same stimulus parameters) for the ON- (left; n = 67) and OFF (right; n = 54) cell population. This response variability is typically <40 ms for both the ON- and OFF-cell populations.

RGCs respond to repetitive visual stimuli with a series of light responses whose potential inconsistency might be responsible for some of the above-observed wide range of RGC response delays^24^. To examine this possibility, we determined the variability of light evoked response delays for a cohort of randomly selected RGCs (n = 94). The trial-to-trial variability of responses varied across the RGC population (Fig. 2a). Some cells showed rather high variance in response delay values, whereas others displayed higher fidelity with very low trial-to-trial variance (Fig. 2b). The trial-to-trial variability of ON- and OFF-response components were overall very similar and did not differ significantly (p = 0.54; Mann-Whitney test). According to our distribution histograms, most RGCs displayed a rather low response delay variability (ON-cells: mean = 21.7 +/− 20.1 ms (SD), n = 63; OFF-cells: mean = 23.8 +/− 25.9 ms (SD), n = 53) and only a few cells possessed delay ranges appearing high enough to overlap with the previously observed wide response delay range. These observations therefore indicate that trial-to-trial variability contribute little (if any) to the broad, population level response delay range.

### RGC response delays are subtype specific

**Figure 3:**
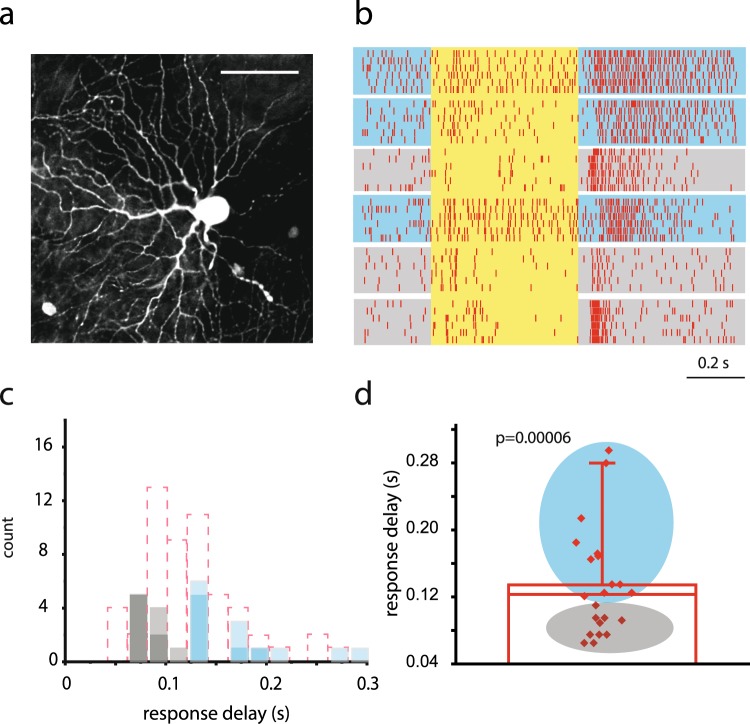
Subtype Specificity of RGC Response Delays. (**a**) Image showing a Neurobiotin injected RGC with a typical OFF-alpha morphology. Scale bar 50 μm. (**b**) Raster showing photopic light responses of 6 RGCs with clear OFF-alpha cell morphology; 6 consecutive responses were recorded for each cell. Response kinetics of our OFF-alpha RGCs could be clearly subdivided into brisk-transient (marked with grey shading) and sluggish-sustained (marked with light blue shading) physiological subpopulations supporting previous reports^26,29^. (**c**) PSTH based response delays were split into brisk (gray) and sluggish (blue) populations (for comparison, all Tungsten electrode recorded OFF-RGC delays are shown in pink dashed bars in the back). Data of Tungsten electrode recorded OFF-alpha RGCs are highlighted, whereas those of cell-attached patch recordings are shown with 50% opacity. This histogram shows that a single RGC subtype (either the brisk-transient or the sluggish-sustained OFF-RGCs) occupies only a narrow latency range. (**d**) When an average OFF-alpha RGC response delay was determined, all brisk-transient cells displayed below-average (grey) latencies, whereas those of sluggish-sustained cells (light blue) were above average. A statistical analysis resulted in a significant difference (p = 0.00006; Mann-Whitney test) between sluggish and brisk OFF-alpha cell responses.

The relative high fidelity of response delays observed in individual RGC recordings suggests that response latency is subtype specific. In this scheme, the observed broad range of response delays was independent of trial-to-trial variability (as shown above) but rather reflected the heterogeneous nature of our dataset comprising many different RGCs with subtype specific response delays. To test this hypothesis, we targeted OFF-alpha RGCs in the mouse retina that could be readily discerned in the DIC view based on their large spherical or somewhat elongated soma and the rather eccentric nucleus position^25–28^. It has been reported that the population of OFF-alpha RGCs are comprised by two subpopulations with either brisk transient or sustained response profiles^29–33^, therefore we expected to obtain two corresponding populations of recordings with distinguishable response speeds in our dataset. Subsequent Neurobiotin injections confirmed the identity of OFF-alpha RGCs, based on well-established soma/dendritic morphological criteria including the presence of stout and generally smooth primary processes, stratification of dendrites in sublamina-a, relatively large dendritic arbors (~185 μm in diameter) as well as the characteristic tracer coupling pattern (both homologous coupling to first tier neighbor OFF-alpha RGCs and heterologous coupling to nearby amacrine cells^25–28^). Only cells with clear OFF-alpha morphology were used in the following analysis (Fig. 3a). As expected, the above-mentioned physiological distinction was apparent in our recordings, where RGCs identified with clear OFF-alpha morphology (n = 14) showed either transient or sustained response characteristics in our sample (Fig. 3b). Furthermore, transient cells appeared to have shorter time-to-peak latencies than those with sustained responses (Fig. 3b). In addition to extracellular recordings, patch electrodes (R = 6 MΩ) were also utilized to carry out OFF alpha cell spike recordings in cell-attached mode (n = 9). Subsequent rapture of the membrane and dye injections confirmed the morphological identity of all recorded cells. The collected dataset, similar to those above, resulted in sluggish (n = 6) and brisk (n = 3) subpopulations, thus indicating that the physiological division of the RGC population is independent of the applied method. Next, a combined distribution histogram was created to compare OFF-alpha RGC response latencies to those of all other OFF-cells in this study. This clearly divided our targeted cells into two populations with either brisk-transient or sluggish-sustained responses (Fig. 3c,d) with statistically separable response delays (brisk-transient: n = 10, mean delay = 87 ms +/− 1.8 SD; sluggish-sustained: n = 13, mean delay = 177 ms +/− 5.8 SD; Mann-Whitney test, p = 0.00006) Thus, our cells undoubtedly corresponded well to those of transient and sustained OFF-alpha cells in previous reports. These latter experiments demonstrated that response latencies were subtype specific in the case of the two OFF-alpha RGC populations, further suggesting that all RGC response delays are subtype specific.

### RGC response delays are determined by upstream retinal circuits

**Figure 4:**
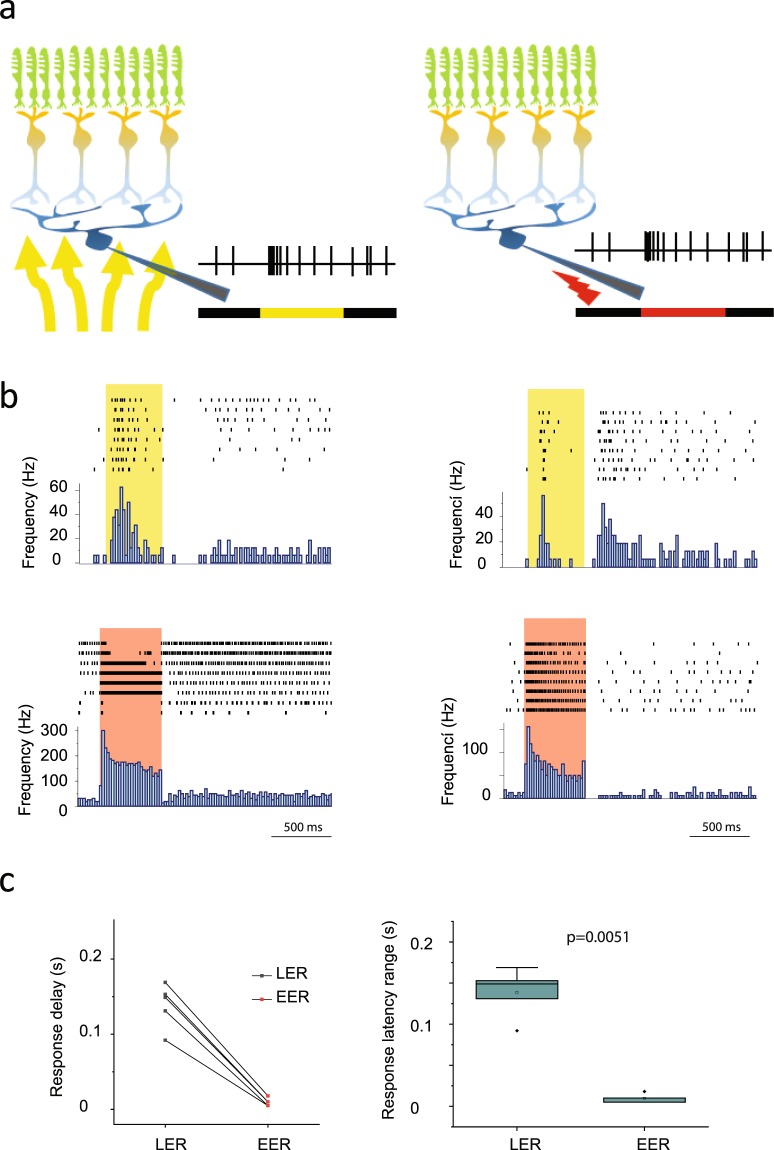
The RGC Response Delay Range Considerably Decreases when Upstream Retinal Circuits are Bypassed. (**a**) Schematic drawings depict the two recording paradigms; classical loose-patch extracellular recording with a glass pipette (left) upon light stimulation (yellow arrows and yellow trace) and recordings following an electrical stimulation with a current pulse (red lightning sign and red trace). (**b**) Individual responses of two representative RGCs (left and right panels) are shown by the raster plots, whereas mean responses are reflected by the PSTHs to light-(upper) and electrical stimulation (bottom), respectively. Histogram displays the reduction of response delays when stimuli were switched from light- (top panels) to electric stimulation (bottom panels) for the examined RGCs (n = 5). (**c**) Besides shorter latencies, electrical stimulation of RGC somata resulted in a statistically significant shrinkage of the latency range (by subtracting response delays of the slowest and fastest responses of each cell) for the examined cell population (p < 0.05; paired t-test).

One may speculate that a subtype specific difference in response delays is due to dissimilar membrane properties, rather than differences in signaling speeds through retinal streams to RGCs - or the combination of both. One way to examine the contribution of the upstream retinal network in response delays is to stimulate RGCs with and without the upstream retinal circuitry and compare the results. If the response delay variety persists when the retinal circuitry is bypassed, then response latency is determined by dissimilar membrane properties rather than different signaling speed *via* the presynaptic retinal network. To this end, we recorded the RGC spike output (n = 5) first by stimulating retinas with a full-field photopic light flash (I = 7000Rh*/rod/s) and then we maintained the preparation in dark and switched to a direct electrical stimulation of the target RGC soma. To standardize electrical stimulations, recordings of this experiment were carried out by glass micropipettes in a cell-attached mode (R = 6 MΩ). RGC macro-seals were maintained on a stable membrane potential (V_m_ = −60 mV) and stimulating impulses were applied through the recording pipette (+100 mV step for 0.5 s; Fig. 4a). PSTHs of both light- and electrically stimulated responses were collected and compared (each PSTH was generated upon a minimum of 6 responses for both light and electrical stimulation tests). Each examined RGC clearly displayed shorter response delays following electrical stimulation when compared to light evoked counterparts (Fig. 4b,c). Therefore, as expected, RGC responses appeared quicker when stimulated electrically in the absence of the upstream neuronal network and the corresponding synaptic delays. Moreover, when response delay ranges (subtraction of shortest and longest delays for each cell) of each RGC appeared considerably smaller for electrical stimulation than as a response to light stimulation (Fig. 4c; p = 0.0051; paired t-test). Thus, this indicates that bypassing the retinal circuitry (besides making it faster) decreases response variability including response delay. Both individual responses of the same cell and responses across the examined population appeared to show very similar response delays (Fig. 4c). This suggests that RGC response latency is not determined by membrane properties (that can be very different for various cell types), but rather determined by the different signaling speed of the presynaptic retinal network.

**Figure 5:**
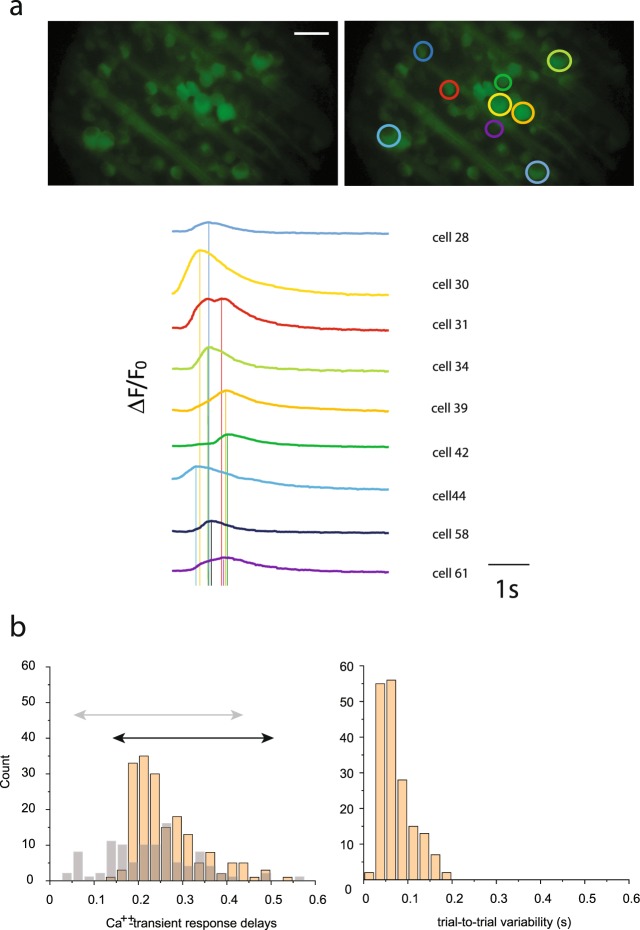
Light Evoked RGC Ca^++^ Transients Display a Broad Range of Latencies. (**a**) Image shows RGCs in the whole-mount Thy1-GCaMP3 mouse retina without (top left) and with (top right) the ROIs of selected RGCs. Traces to the right show light evoked Ca^++^-transient events for the color coded RGCs (bottom). (**b**) Histogram shows the response delay distribution of light evoked Ca^++^ -transient events (left) and trial-to-trial variations (right) for many RGCs (n = 178) in the examined Thy1-GCaMP3 samples. Gray bars in the back show a similar response delay distribution histogram for responses of n = 102 RGCs recorded in the PV-GCamP6 mouse line. Although RGCs from this latter line displayed overall faster response kinetics, the delay distribution histogram is just as broad as the one observed for RGCs recorded in the Thy1-GCaMP3 line (represented by the colored and grey arrows on the top for GCaMP3 and GCaMP6 mice, respectively), justifying that the broad range of response delays is not due to the slow kinetics of the GCamP3. Scale bar: 50 μm.

Another set of supporting results was obtained in our Ca^++^-imaging experiments in the Thy1-GCamP3 mouse retina (Fig. 5a; n = 178; full-field 500 ms light steps, I = 7000Rh*/rod/s, λ = 488). Although the speed of a RGC Ca^++^-transient is considerably slower than the corresponding spikes and RGC spikes rather occur at the steepest rising phase of a particular Ca^++^-transient not at the peak (based on our own unpublished observations - not shown), their dynamic response range has been reported as adequate under natural stimulus conditions^34^. Therefore, we examined the range of response speeds by utilizing this methodology as well. Note that slow responses were not the results of the slow kinetics of the GCamP3 construct as the faster GCamP6 indicator resulted in both slow and fast responses when parvalbumin specific GCamP6 expressing RGCs were examined (Fig. 5b; n = 102). A broad range of time-to-peak delays of RGC Ca^++^-transients would suggest that RGC EPSCs (represented by the detected Ca^++^-transient events) are already heterogenous in terms of their latencies, thus they spread out in time similar to PSTH time-to-peak values. In fact, we found that examined RGCs displayed Ca^++^-transients with a broad range of delays (140–520 ms; mean = 261.8 +/− 76.4 ms SD) and that was clearly distinguishable from the trial-to-trial response variation range detected for individual cells (mean = 73.46 +/− 36.8 ms (SD); Fig. 5b). Therefore, we found that besides spike outputs, RGC slow potential responses varied considerably as well. This strongly suggests that a possible variation in spike generating thresholds is not critical in maintaining the observed great response latency range.

### Both light evoked Ca^++^-transients and RGC spiking are altered by pharmacological blockade of retinal signaling streams

**Figure 6:**
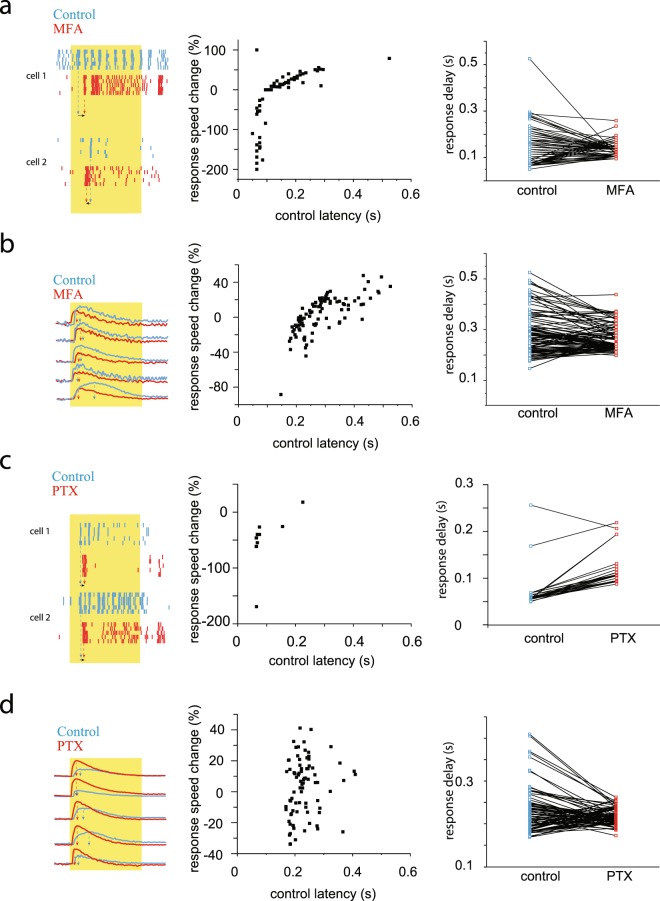
Lateral Inhibition and GJ Mediated Signaling Considerably Alter RGC Response Delays. *(***a**) Representative RGC responses (to the left) in control conditions (blue) and after a GJ blockade (red; MFA 40 μM concentration). Histogram in the middle displays MFA induced changes of PSTH-based response delays for the examined RGC population (n = 159; ON/OFF:107/52). Diagram to the right shows a reduction in the RGC response delay range due to MFA application. Response delay changes were calculated as (delay_control_-delay_mfa_)/delay_control_ * 100). (**b**) RGC light responses in control conditions (blue) and after a GJ blockade (red; MFA 40 μM concentration; left panel). Histogram in the middle displays MFA induced changes of Ca^++^-transient light response delays for the examined RGC population (n = 95); and diagram to the right shows the reduction of RGC response latency range following MFA application. (delay_control_-delay_mfa_)/delay_control_ * 100). (**c**) Representative RGC responses in control conditions (blue) and under the blockade of GABA-ergic signaling (red; PTX 50μM concentration). Histogram in the middle displays PTX induced changes of PSTH based response delays for the examined RGC population (n = 31). Diagram to the right shows the GABA blockade induced changes of RGC response delays and also the reduction of the delay range induced by PTX application. Response delay changes were calculated as (delay_control_-delay_ptx_)/delay_control_ * 100). (**d**) RGC Ca^++^-transient light responses in control conditions (blue) and after following PTX incubation (red; PTX, 50 μM concentration). Histogram displays PTX-induced changes in Ca^++^-transient light response delays for the examined RGC population (n = 83; middle panel), while the diagram to the right shows the reduction in the latency range induced by the pharmacological intervention. Response delay changes were calculated as (delay_control_-delay_ptx_)/delay_control_ * 100.

The previous results strongly suggest that the great variety in subtype specific response delays is due to the dissimilar speeds by which signals reach RGC targets *via* parallel retinal pathways. To see if this variety in signaling speeds is due to active fine-tuning performed by retinal interneuron circuits, we performed both spike recordings and Ca^++^-imaging measurements during pharmacological blockade of specific circuit elements including GABA-ergic inhibition and signaling through gap junctions (GJs). GJ blockade was carried out by the addition of meclofenamic acid (MFA; 40 μM) into the superfusate and light evoked RGC activity was recorded prior to, and following application. The GJ blockade induced changes in the response delay for all examined RGCs, either by decreasing or increasing response speeds. This dichotomy in latency change was observed in both spike recordings and Ca^++^-transient signals and it was as high as 47% and 200%, respectively (Fig. 6a,b). Although, the effect of this GJ-blockade varied considerably across the examined RGC population in both polarity and extent, the overall range of RGC response delays decreased from 50–300 ms (~250 ms) to 75–270 ms (~195 ms) in spike recordings and from 150–530 ms (380 ms) to 180–350 ms (170 ms) in Ca^++^-imaging experiments.

GABA receptor blockade (mixed A- and C-type) was achieved by incubating retinas in 50 μM picrotoxin (PTX) and recordings were performed both under control conditions and following drug application. Similar to results obtained under GJ blockade, GABA receptor inactivation resulted in either an increase or a decrease of response latency for most examined RGCs, irrespective of the experimental approach (Fig. 6c,d). However, the range of RGC response latencies dropped tremendously when GABA-A and -C receptors were inhibited pharmacologically. The response latency range dropped from 60–225 ms (~165 ms) to 90–195 ms (~105 ms) in spike recordings and from 170–420 ms (~250 ms) to 175–275 ms (~100 ms) in Ca^++^ -imaging experiments (Fig. 6c,d).

### Stimulus parameters significantly determine RGC response delays

**Figure 7:**
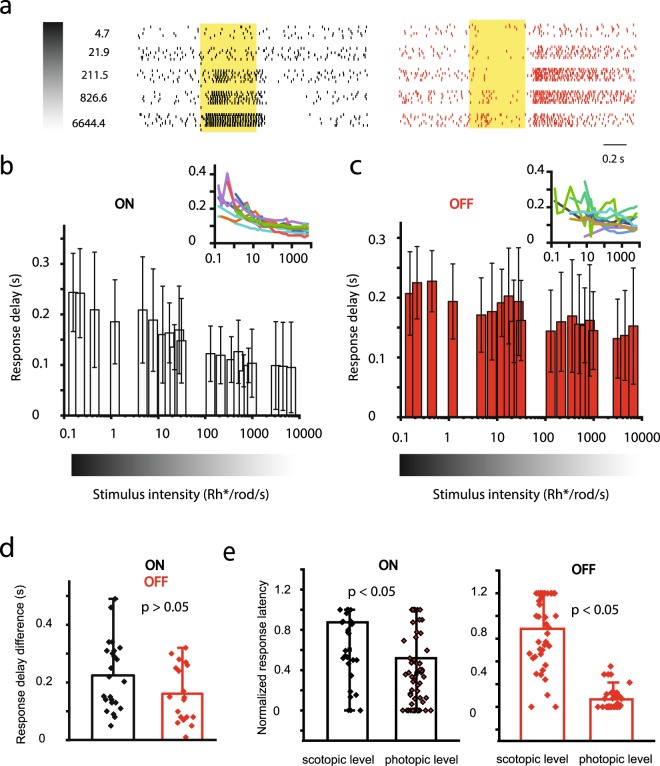
RGC Response Delays Vary with Stimulus Strength. *(***a***)* Raster diagrams display 6 consecutive light responses of an ON-(left) and an OFF-RGC (right) evoked by stimuli with an increasing strength (numbers to the left reflect light intensity values in Rh*/rod/s). (**b**,**c**) Bar graphs display average response delays of the examined ON-(**b**) and OFF-(**c**) RGC population as a function of stimulus intensity (x axis, Rh*/rod/s; insets show delay values for individual RGCs). (**d**) Bar graph showing maximum response delay differences for the ON- and OFF-RGC subpopulation. Whereas OFF-cells seemed somewhat insensitive to the change of the stimulus strength, ON-cell responses displayed a stimulus strength-dependent decrease of the response latency (increased speed). However, this apparent difference in ON- vs. OFF-RGC signaling was not statistically significant (p = 0.068; Mann-Whitney test). (**e**) Bar graphs show a comparison of response latencies to scotopic (I = 0.15–1.2 Rh*/rod/s) vs. photopic (I = 978–6645 Rh*/rod/s) light stimuli for the same set of RGC population. Photopic light responses were significantly faster for both ON (p = 0.0045; Mann-Whitney test) and OFF cells (p << 0.001; Mann-Whitney test).

It has been shown that spatiotemporal stimulus features affect response latencies and differential spike latencies can be utilized by the brain to encode details of the visual scene^18,24,35–37^. Here, we tested how further non-spatiotemporal stimulus parameters, including stimulus strength as well as chromatic features, affect RGC response delays. First, the effects of stimulus intensity changes were tested by performing RGC (n = 16) recordings while retinas were excited with a series of full-field light stimulus steps with increasing strengths (Fig. 7a; I = 1–7000 Rh*/rod/s; λ = 488 nm; scotopic to photopic). Time-to-peak delay values of responses were obtained and corresponding PSTHs were generated separately for each light step. In general, RGCs responded with gradually decreasing delays as the intensity of the light stimulus was increased from high scotopic (~1 Rh*/rod/s), to high photopic (~7000 Rh*/rod/s; (Fig. 7a–d)). This inverse relationship between stimulus intensity and RGC response delay was evident in most examined cells but it was clearly more pronounced in the ON-cell population. Although some OFF-cells deviated from this latter behavior, when all OFF-RGCs were grouped together the inverse stimulus strength/response delay relationship could be clearly detected (Fig. 7c). This was even more obvious when response delays obtained for faintest and brightest stimuli for each RGC were first subtracted and then difference values were compared (Fig. 7d). In this latter comparison, OFF-cell delay changes showed less stimulus dependency than ON-cells (ON: mean = 224.4 ms, SD = 120 ms; OFF: mean = 160.9, SD = 97.9), but this observed ON/OFF difference was not statistically significant (p > 0.068; Mann-Whitney test). We also performed a second analysis on the same set of RGC recordings in which light responses were first normalized (throughout the entire set of light stimuli) and then scotopic (I = 0.15–1.2 Rh*/rod/s) and photopic (I = 978–6645 Rh*/rod/s) responses were compared. By comparing RGC response latencies after this normalization photopic responses appeared significantly shorter for both ON (p = 0.0045) and OFF RGCs (p << 0.001). This analysis thus unequivocally showed that response delays are in fact subjects of stimulus intensity.

**Figure 8:**
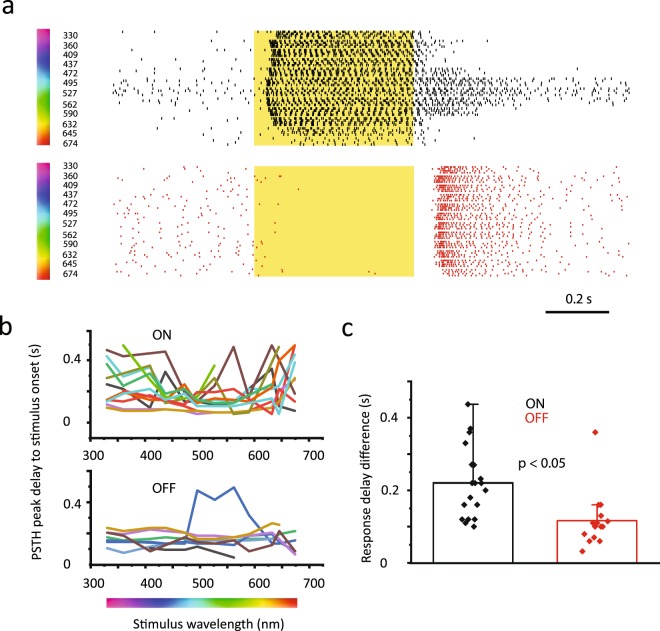
RGC Response Delays Vary with Spectral Features of Light Stimuli. (**a**) Raster diagrams show representative ON-(top) and OFF-(bottom) RGC responses to photopic light stimuli of various spectral lengths (wavelengths are shown by the number to the left; six trials for each stimulus). Clearly, response delays were prone to stimulus wavelength changes. (**b**) Histogram displays variations of response delays (y axis) of individual RGCs (color coded) as a function of stimulus wavelength (x axis). ON-RGCs (top graph) seemed to be more sensitive to stimulus wavelength changes than those of OFF-RGC (bottom graph) counterparts. (**c**) Bar graph showing maximum response delay differences for both ON- and OFF-RGC responses. ON-cells showed significantly larger variation in the response delays than the examined OFF-RGC population (p = 0.0004; Mann-Whitney test).

Next, effects of chromatic changes of the stimulating light in RGC response delays were tested. Light evoked responses of randomly selected RGCs (n = 34) were recorded and compared while the wavelength of the stimulating full-field light beam was gradually increased through 12 wavelength steps from 330 nm to 674 nm (330, 360, 409, 437, 472, 495, 527, 562, 590, 632, 645 and 674 nm; Fig. 8). While most examined RGCs displayed some changes in their response latencies, they differed considerably in terms of the extent of latency changes induced by the chromatic alteration of visual stimuli. One observation was that OFF-cell response delays in general were less prone to spectral changes of the stimulus (Fig. 8c). This difference was quantified by comparing greatest response delay differences of ON (n = 19) and OFF (n = 15) RGCs by subtracting the shortest measured delay values from the longest for each cell. Mean response delay differences were 221 (SD = 97.8 ms) and 116 ms (SD = 76.2 ms) for ON- and OFF-cells, respectively. This observed difference was statistically significant (p < 0.00039; Mann-Whitney test). Another, somewhat expected observation was that the linear change in the light wavelength induced a non-linear change in RGC response delays. This phenomenon was clear in both the spike recordings and the response latency/wavelength functions of individual RGCs (Fig. 8b). While most RGCs displayed the shortest response delay evoked by green light (the 495 and 527 nm steps), some cells exhibited a secondary latency minimum as well evoked by the 360 nm UV-light beam (Fig. 8a,b). The above experiments clearly indicate that various stimulus parameters, like strength and wavelength, considerably alter RGC response delays.

## Discussion

### Methodological considerations of latency measurements

One of the critical issues of the present work is the accuracy of latency measurements and whether their errors distort the interpretation of the data. As most experiments were carried out without visual targeting, recorded RGCs formed a heterogenous population in terms of their response latencies. In fact, all samples contained neurons with brisk-, sluggish- and intermediary responses as well as cells with no preference for any response phenotype regardless of the hour of the day, the age of the mouse (P_20_-P_90_ mice were used) or the freshness of the preparation (RGCs were recorded for up to 6 hours with no change in response characteristics). Therefore, as for the validity of our measurements, we are confident that the observed great variety in response delays is not deceptive and unrelated to experimental circumstances. In fact, similar latency differences have been reported extensively in retinas of various species^12,18,35^ and thus the data presented here further support these previous findings.

Another methodological aspect is the time resolution by which events (action potentials) are collected to obtain PSTHs to determine latency values. In this study, we utilized a 10 ms bin size to generate PSTHs, which inherently create a +/−5 ms error in the dataset. However, this is negligible when compared to the observed latency range (~40–240 ms) across the mouse RGC population. Therefore, measurement error could not contribute considerably to the detected latency range.

**Figure 9:**
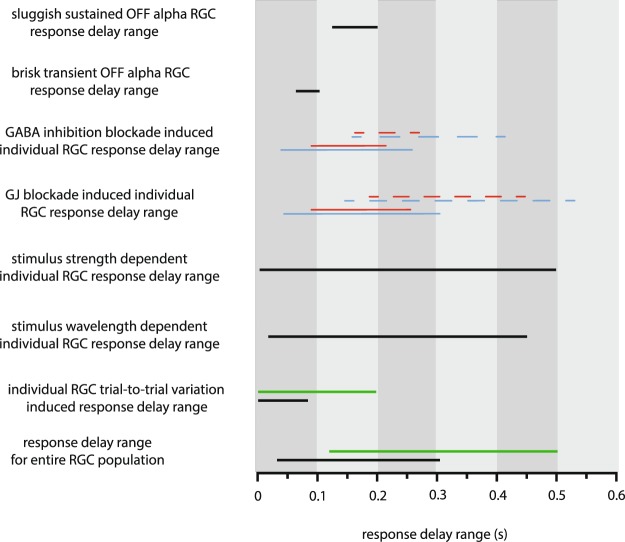
Summary of Response Latency Ranges in Various Stimulating Circumstances. Diagram displays a comparison for response latency variations and changes observed in this study. Stimulating and recording paradigms are shown to the left, delay ranges are represented by horizontal bars (black - extracellular recordings, green - Ca^++^-imaging experiments, blue and red – control and pharmacological conditions for extracellular recordings (solid lines) and Ca^++^-imaging experiments (dashed lines)).

A third aspect is the relevance of RGC spiking frequency (and derivative PSTHs) in terms of the code sent towards visual brain centers. In fact, there has been a long debate concerning the nature of the neuronal code utilized by the brain to perform a variety of tasks. The spiking rate as a parameter that the brain might utilize to decode incoming information from sensory organs is just one of the several possible variables^38,39^; reviewed by^35^. Here we showed evidence that first spike latencies and PSTH peak latencies of the same RGC population, though they differed in their actual values, displayed comparably great ranges. In addition, we also showed that the observed broad response latency range was present in our experiments regardless of the data acquisition method (Tungsten extracellular recording, MEA extracellular recording, Ca^++^-imaging; Fig. 9). Therefore, we conclude that neither methodological nor conceptual errors contributed significantly to our findings, indicating that RGC response latencies bear with physiological meaning regarding the coding of visual signals.

### The range of latency differences among parallel retinal pathways

We found that RGC signal latencies varied on a rather broad range of 40–240 ms (Fig. 9). Sinha and colleagues^40^ have reported recently that foveal photopic signaling of midget RGCs endures a 30 ms delay compared to peripheral counterpart midget cells in the primate retina. This phenomenon was accounted to structural differences of foveal vs. peripheral cone photoreceptors. As the mouse retina holds no retinal specializations (area centralis, visual streak or fovea) and displays only little centro-peripheral structural differences^21,41^, retinal locations of recorded RGCs could not be the basis for latency differences observed in this study. Note that the mouse retina displays a dorso-ventral asymmetry in terms of cone photoreceptor chromatic sensitivity^42–44^. However, we recorded from both slow and fast responding RGCs in all retinal locations, ruling out the possibility that this dorso-ventral difference plays a role in the observed distinction. Moreover, the finding in the primate retina was based on responses of one particular cell type, the midget RGC, whereas we presented recordings from many different RGC subtypes in the mouse (also note that there are no midget RGCs in the mouse retina). In addition, the latency range observed here was about an order of magnitude broader than those reported for primate midget cells. Therefore, the basis for our findings must reside elsewhere in the mouse retinal circuitry.

We presented evidence showing that the RGC trial-to-trial response variability may contribute up to 10% (~24 ms) to the observed broad latency range. However, much of the observed variability must be accounted for by other mechanisms. We also showed through two example RGC subtypes that their responses covered only narrow latency subranges. This indicates that each mouse RGC subtype contributes with a narrow subrange to the detected population range. Such subtype specific response latency differences can be defined by dissimilar RGC membrane properties or by distinct signaling speeds of intraretinal circuits targeting each of the RGC subtypes. In a series of experiments using the dual electrical/light stimulating paradigm, we showed that RGCs in fact respond to extracellular electrical stimuli within the first PSTH bin (<10 ms), regardless of the recorded RGC subtype. Moreover, the range of response latencies dropped from ~200 to ~10 ms when light stimulation was replaced with electrical stimuli. Thus, bypassing the retinal circuitry diminished the broad range entirely, indicating that the population-wide broad latency range is determined primarily by the various speeds of RGC targeting intraretinal circuits.

Certainly, some RGCs studied here are subtypes that send output to non-image forming brain centers in the midbrain and thus the latency by which they operate does not necessarily have to match those that project to the dorsal lateral geniculate nucleus and eventually the visual cortex. However, we suspect that most of our recorded cells do contribute to visual image formation and thus certain visual features travel faster to the cortex than others. In fact, psychophysiological experiments showed that object movement is perceived faster than object contour, contrary to the coincidence, or even the opposite order of the two types of stimuli^15–17^. It is possible that vital visual features, such as movement (e.g. image of a moving predator), are carried faster to induce the necessary visually driven behavior well before contours, color or other features of the same object perceived.

### Signaling speed is fine-tuned by elements of upstream retinal circuits

Our main findings were based on the spike output of mouse RGCs but at the same time, they were also confirmed by Ca^++^-imaging measurements. Although transient Ca^++^-responses were several times slower than the PSTH time-to-peak latency values, their latency range was similarly broad (Fig. 9). This indicates that stimulus induced depolarizations (and Ca^++^-transients) are already heterogenous regarding their latencies, further suggesting that the upstream presynaptic circuitry provides inputs with heterogenous speeds to RGC subtypes. We found in both electrophysiological and imaging experiments that blocking either inhibition or electrical synapse-mediated signaling alters RGC response latencies. While the blockade of these circuit elements resulted in a mixture of effects (either a decrease or an increase) on response kinetics of individual cells, the population wide RGC latency range shrank in both cases (Fig. 9). This indicates that actions of various circuit elements, such as GABAergic inhibition or signaling through electrical synapses (shown in this study), serve to diversify RGC response latency values evoked by a certain stimulus.

### Function of latency changes induced by altered stimulus parameters

It has been previously reported that the visual system informs the brain about different visual aspects through varying signaling speeds^45^. For example, foveal photopic pathways of the primate retina deliver slow but high acuity signals about fine structural details to the brain, whereas peripheral retinal pathways convey rapid signals essential for quick vision-driven motor activity^40,46^. This indicates that the shared labor to signal all aspects of the visual scene towards brain centers occurs via a variety of speeds which manifests as a series of distinct, subtype specific RGC response latencies in this work. In addition, RGC response latency has been shown to be subject to fine changing of visual features^18,35,36^. This indicates that the RGC response latency is readjusted by any slight variation in a certain visual feature and conveyed towards the brain at altered speed. Response delays of V1 simple cells, for example, can be altered with 40–50 ms as a response of the modulation of contrast or orientation of bar- or drifting sign-wave stimuli^47–49^. Here we present further evidence for such latency-based signal distinction by showing that RGC response latency switches when stimulus color or strength is modulated. However, this phenomenon must come with functional consequences: incoming inputs can only be bound together by final integrators in visual brain centers if they fit in the integration time window. Out of sequence or uncorrelated inputs relative to those carried by other feature detector signaling pathways might be dissipated entirely or could have a lower weighed effect in forming the output signal. On the other hand, inputs that come in the right sequence can reinforce each other and have a greater effect on the output (note that not necessarily the fastest signals are correlated with other converging signals). It has been reported that the dynamics of postsynaptic integration are in fact strongly dependent on incoming spike times^50^. This, together with the evidence of ~2–9 ms time constant measured for cortical pyramidal cells in the cat^51^, suggests the existence of a strong temporal filter in the visual cortex. This filter utilizes afferent signals correlated on a narrow temporal scale (<10 ms) to generate the spike output and dumps the rest, thereby filtering out non-adequate signal components. Based on the above converging evidence, we hypothesize that the ability to slightly alter response latency is a tool exploited by the visual system to weigh incoming signals and fine-tune visual perception. Moreover, key neuronal and synaptic components of this latency modulating mechanism are embedded in the retinal hyper-circuitry.

## Supplementary information

Supplemental Figures 1 and 2
