# Supplementary material for: Response Latency Tuning by Retinal Circuits Modulates Signal Efficiency
Source: Sci Rep. 2019 Oct 22;9:15110. doi: 10.1038/s41598-019-51756-y (PMC6806000; doi:10.1038/s41598-019-51756-y)
Supplement: Supplementary file 1 — Supplemental Figures 1 and 2 [file 41598_2019_51756_MOESM1_ESM.pdf]

Response Latency Tuning by Retinal Circuits Modulates Signal Efficiency

Ádám Jonatán Tengölics, Gergely Szarka, Alma Ganczer, Edina Szabó-Meleg,

Miklós Nyitrai, Tamás Kovács-Öller, Béla Völgyi

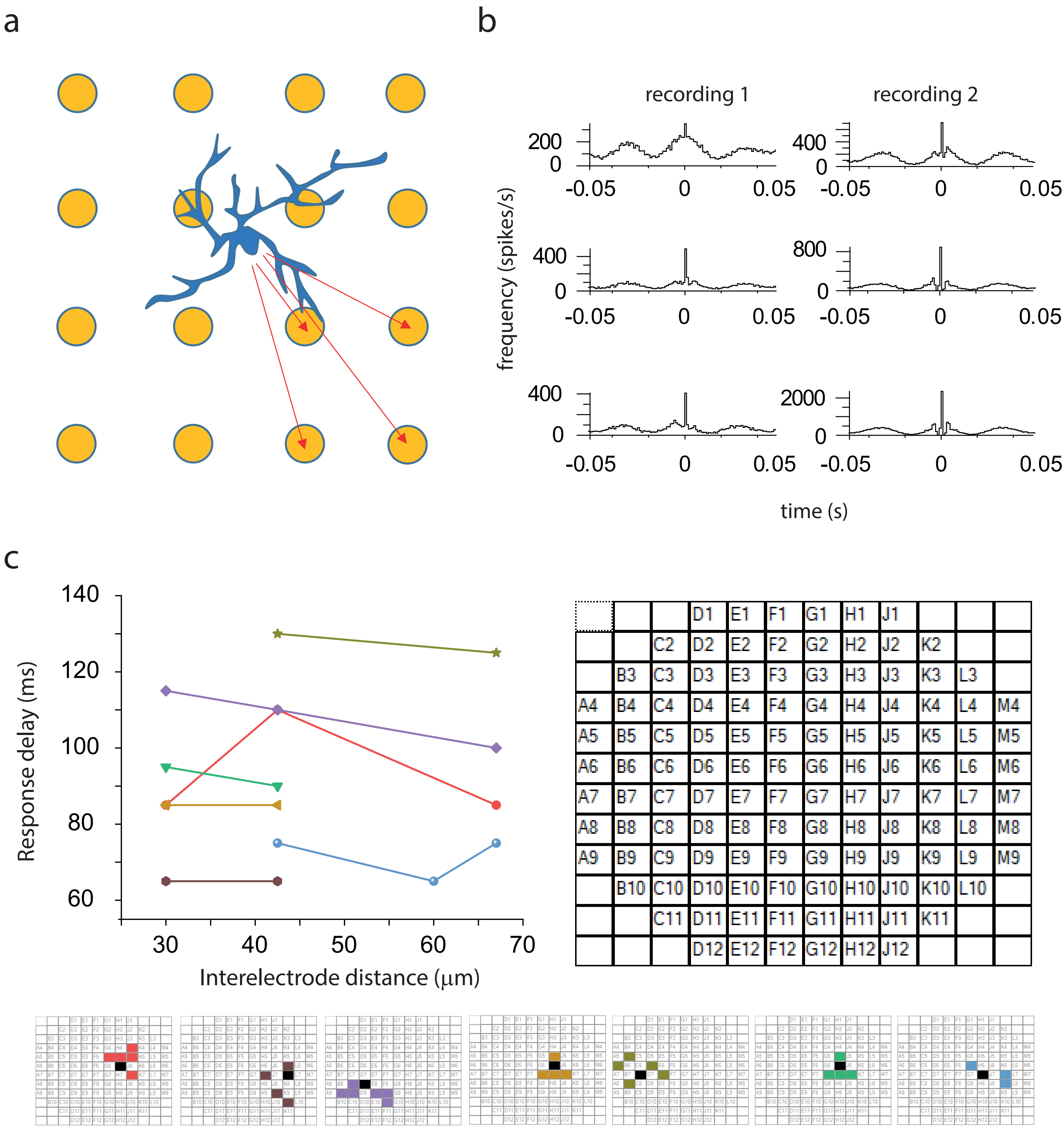
a
b
recording 1
recording 2
200
0
400
0
800
2000
-0.05
0
0.05
frequency (spikes/s)
time (s)
c
Response delay (ms)
140
120
100
80
60
30
40
50
60
70
Interelectrode distance (μm)
D1 E1 F1 G1 H1 J1
C2 D2 E2 F2 G2 H2 J2 K2
B3 C3 D3 E3 F3 G3 H3 J3 K3 L3
A4 B4 C4 D4 E4 F4 G4 H4 J4 K4 L4 M4
A5 B5 C5 D5 E5 F5 G5 H5 J5 K5 L5 M5
A6 B6 C6 D6 E6 F6 G6 H6 J6 K6 L6 M6
A7 B7 C7 D7 E7 F7 G7 H7 J7 K7 L7 M7
A8 B8 C8 D8 E8 F8 G8 H8 J8 K8 L8 M8
A9 B9 C9 D9 E9 F9 G9 H9 J9 K9 L9 M9
B10 C10 D10 E10 F10 G10 H10 J10 K10 L10
C11 D11 E11 F11 G11 H11 J11 K11
D12 E12 F12 G12 H12 J12

Supplemental Figure 1. ***Soma to Electrode Distance does not Contribute Significantly to RGC Response Latency. a.*** Schematic drawing illustrates positions of recording electrodes (yellow spots) relative to the soma of a recorded RGC. Various arrow lengths represent differences in the distance (and time) currents need to travel in the extracellular space to be detected by each electrode. ***b.*** Cross-correlation functions (CCF) show sharp peaks at time 0 indicating that selected electrodes recorded spikes of the same RGC. CCF triplets in the same column display spike correlations detected by three nearby electrodes while spike events of a fourth electrode were used as a reference. ***c.*** Histogram showing response latency values for n=7 RGCs as a function of soma-to-electrode distance (top left). Matrix shows the electrode arrangement in a typical 64 channels MEA recording (top right). Color coded electrodes (bottom) depict recording sites for RGCs whose response latencies appear in the top left panel with the same color-code. In each recording, the centermost electrode (black) was assumed to be in the vicinity of the recorded RGC soma (distance=0), electrode distance was 30 μm, and latencies measured by 2 or more electrodes in the same distance were averaged.

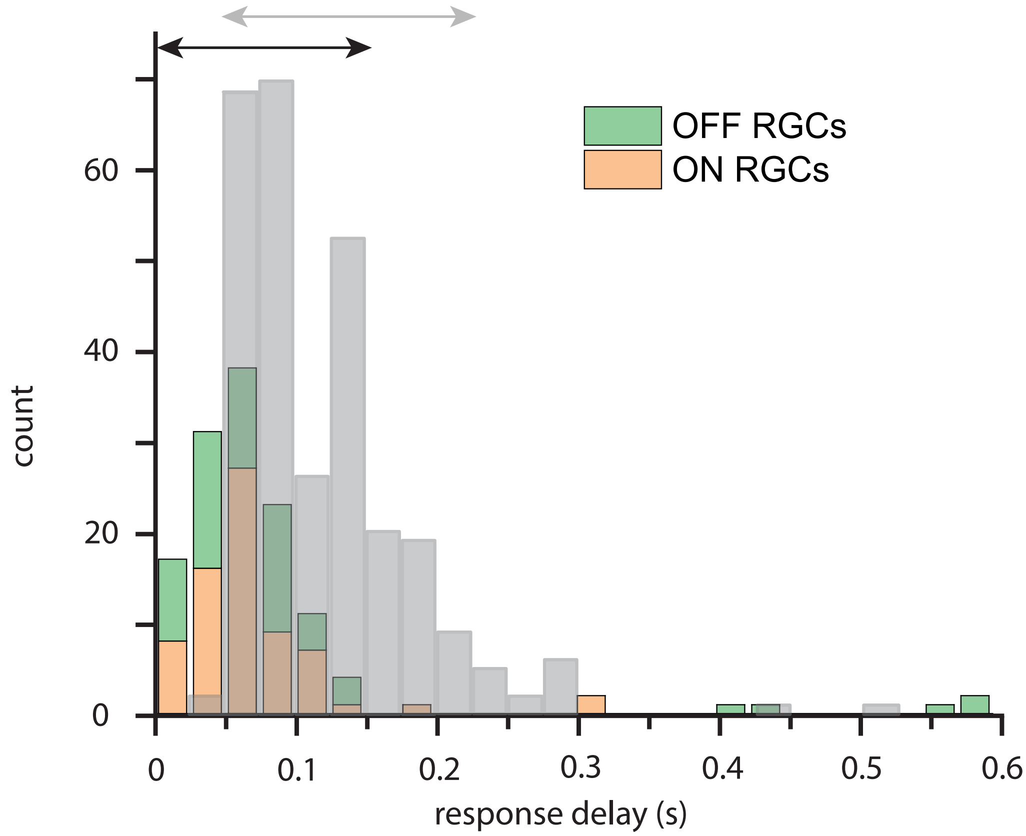

OFF RGCs
ON RGCs
60
40
20
0
count
0
0.1
0.2
0.3
0.4
0.5
0.6
response delay (s)

Supplemental Figure *2. RGC First Spike Delays Cover a wide range.* First spike delay values were characterized, and their distribution is shown in this diagram (highlighted bars). For comparison, PSTH peak delays for the same RGCs are shown in the back (gray background). Although, response delay values are lower when determined based on first spike times response delay ranges are similar (represented by the arrows over the bars).
